# Systemic immune reaction in axillary lymph nodes adds to tumor-infiltrating lymphocytes in triple-negative breast cancer prognostication

**DOI:** 10.1038/s41523-021-00292-y

**Published:** 2021-07-05

**Authors:** Fangfang Liu, Thomas Hardiman, Kailiang Wu, Jelmar Quist, Patrycja Gazinska, Tony Ng, Arnie Purushotham, Roberto Salgado, Xiaojing Guo, Sarah E. Pinder, Anita Grigoriadis

**Affiliations:** 1 Cancer Bioinformatics, School of Cancer & Pharmaceutical Sciences, King’s College London Faculty of Life Sciences and Medicine, London, UK; 2 Department of Breast Pathology and Research Laboratory, Key Laboratory of Breast Cancer Prevention and Therapy (Ministry of Education), National Clinical Research Center for Cancer, Tianjin Medical University Cancer Institute and Hospital, Tianjin, China; 3 School of Cancer & Pharmaceutical Sciences, King’s College London Faculty of Life Sciences and Medicine, London, UK; 4 Breast Cancer Now Unit, School of Cancer and Pharmaceutical Sciences, King’s College London, London, UK; 5 Breast Cancer Now Toby Robins Research Center, The Institute of Cancer Research, London, UK; 6 Division of Research, Peter Mac Callum Cancer Centre, Melbourne, Australia; 7 Department of Pathology, GZA-ZNA Hospitals, Antwerp, Belgium

**Keywords:** Breast cancer, Tumour biomarkers, Prognostic markers

## Abstract

The level of stromal tumor-infiltrating lymphocytes (sTILs) in triple-negative (TNBC) and HER2-positive breast cancers convey prognostic information. The importance of systemic immunity to local immunity is unknown in breast cancer. We previously demonstrated that histological alterations in axillary lymph nodes (LNs) carry clinical relevance. Here, we capture local immune responses by scoring TILs at the primary tumor and systemic immune responses by recording the formation of secondary follicles, also known as germinal centers, in 2,857 cancer-free and involved axillary LNs on haematoxylin and eosin (H&E) stained sections from a retrospective cohort of 161 LN-positive triple-negative and HER2-positive breast cancer patients. Our data demonstrate that the number of germinal center formations across all cancer-free LNs, similar to high levels of TILs, is associated with a good prognosis in low TILs TNBC. This highlights the importance of assessing both primary and LN immune responses for prognostication and for future breast cancer research.

## Introduction

Triple-negative (TNBC) and human epidermal growth factor receptor-2 (HER2)-positive breast cancers display higher prevalence of stromal tumor-infiltrating lymphocytes (sTILs) than estrogen receptor (ER)-positive breast cancers^1–3^. The assessment of sTILs at the primary tumor site via light microscopy of haematoxylin and eosin (H&E) stained sections, has been shown to be superior to classical TNM staging in TNBC and HER2-positive breast cancers in predicting outcome^3^, response to chemotherapy^4^, anti-HER2 therapy^5^ and to immunotherapy^6^. Although sTIL assessment is not, as yet, included in national breast cancer pathological minimum datasets, some clinicians are now requesting this information; the aim being to use the data to advise patients on the appropriateness of systemic therapies for example to de-escalate chemotherapeutic regimens in those patients with very high TILs, who have an excellent prognosis. The St Gallen International Consensus Guidelines 2019 for TNBC recommend evaluation of sTILs in these lesions^7^; however, TILs’ scoring should currently not be used to take treatment decisions nor to escalate or de-escalate therapy.

The presence and extent of lymph node (LN) metastasis are associated with shorter disease-free and overall survival in breast cancer^8^, but LNs, as well as being typically the first site of seeding of many solid tumors, also serve as immunological hubs between the tumor and the patient’s systemic immunity. Currently, routine pathological reporting does not extend beyond the assessment of the presence and size of metastasis in the LNs and the presence of extra-nodal extension. Recent immunohistochemical and transcriptional studies have examined the immune context of axillary LNs, reporting qualitative changes in certain immune cell populations, such as an increase of CD68 + macrophages in cancer-free LNs associated with disease progression^9,10^. Based on extensive histopathological analyses of immune and stromal features in primary tumors and axillary LNs, we have previously detailed histological changes in cancer-free LNs that are of value in the prediction of risk of developing distant metastasis^11^. In a series of breast cancers, enriched for TNBC, LN-positive patients with increased germinal center (GC) formation in their cancer-free LNs showed a superior outcome, even compared to LN-negative disease.

In this study, the primary objective was to capture systemic immunity, as identified by histological alterations in cancer-free LNs, and determine whether this carried clinical importance. We conducted an extensive numerical characterization of GC formation in 2,857 involved and cancer-free axillary LNs from 161 TNBC and HER2-positive patients. sTILs and tertiary lymphoid structures (TLS) were also assessed in the primary tumors on standard diagnostic H&E-stained slides^11^. Our secondary objective was to determine whether systemic immune responses would modify the prognostic effect of local sTILs density, indicating that the assessment of the combination of primary and nodal immune response would aid in prognostication.

## Results

### Patient characteristics

**Fig. 1:**
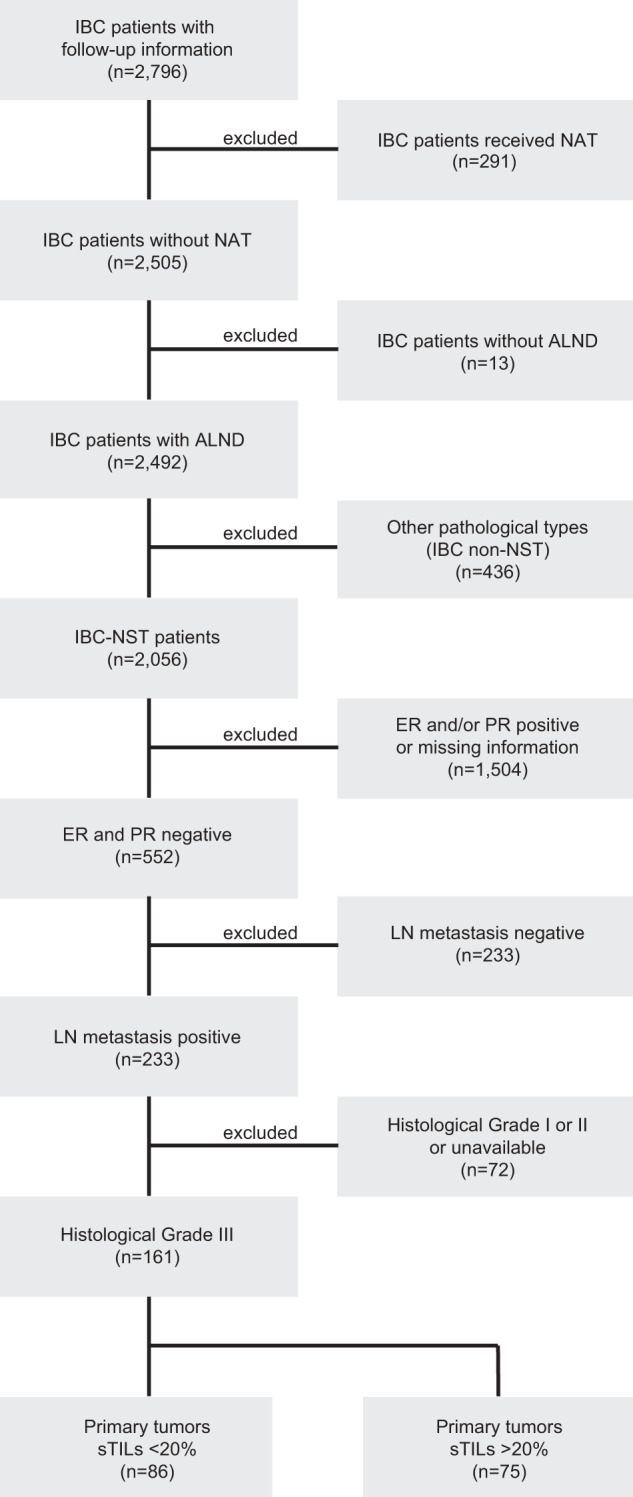
CONSORT diagram. IBC Invasive breast cancer, NAT neoadjuvant therapy, IBC-NST invasive breast cancer of no special type, ER estrogen receptor, PR progesterone receptor, sTILs stromal tumor-infiltrating lymphocytes.

We selected a cohort of patients with invasive breast carcinoma treated between 2005 and 2010 at Tianjin Medical University Cancer Hospital, China, consisting of 161 grade 3 no special type (IBC-NST) HR-negative carcinomas (HER2-positive or TNBC) with positive LNs (Fig. 1). The clinicopathological features of the HER2-positive group were comparable to the TNBC group, with a marginally higher frequency of lymphovascular invasion (79% versus 62%, Chi-squared test, *P* = 0.02) and of higher nodal stage (pN3 27% versus 15%, Chi-squared test, *P* = 0.04) in HER2-positive breast cancer patients (Table 1). For distant disease-free survival (dDFS), median follow-up was 9.08 years (range, 0.92–14.3 years). During follow-up, 34 (21%) patients died of cancer and 47 patients (29%) developed a recurrence, including 17 (11%) local or regional tumor recurrence, and 42 (26%) distant metastases; of these, 70% developed metastasis within the first 3 years after diagnosis (range, 0.16–9.16 years).

**Table 1:** Clinicopathological features and immune features of the primary tumor.

	All cases	HER2	TNBC		sTILs < 20%	sTILs≥20%	
	*n* = 161 (%)	*n* = 62 (%)	*n* = 99 (%)	*P* value	*n* = 86 (%)	*n* = 75 (%)	*P* value
*sTILs*							
<20%	86 (53)	34 (55)	52 (52)		/	/	/
≥20%	75 (47)	28 (45)	47 (48)	0.775^a^	/	/	/
*Tertiary lymphoid structures*							
Absent	123 (76)	46 (74)	77 (78)		72 (84)	51 (68)	
Present	38 (24)	16 (26)	22 (22)	0.602^a^	14 (16)	24 (32)	0.019^a^
*Age at diagnosis*							
<50	66 (41)	20 (32)	46 (46)		35 (41)	31 (41)	
≥50	95 (59)	42 (68)	53 (54)	0.074^a^	51 (59)	44 (59)	0.935^a^
*Tumor size*							
pT1	33 (21)	11 (18)	22 (22)		12 (14)	21 (28)	
pT2	116 (72)	46 (74)	70 (71)		65 (76)	51 (68)	
pT3	10 (6)	4 (6)	6 (6)		8 (9)	2 (3)	
pT4	2 (1)	1 (2)	1 (1)	0.906^a^	1 (1)	1 (1)	0.071^a^
*Histological grade*							
III	161 (100)	62 (100)	99 (100)		86 (100)	75 (100)	
*Lymphovascular invasion*							
Absent	51 (32)	13 (21)	38 (38)		24 (28)	27 (36)	
Present	110 (68)	49 (79)	61 (62)	0.021^a^	62 (72)	48 (64)	0.271^a^
*Lymph node status*							
pN1 (1–3)	90 (56)	27 (44)	63 (64)		47 (55)	43 (57)	
pN2 (4–9)	39 (24)	18 (29)	21 (21)		20 (23)	19 (25)	
pN3 (>=10)	32 (20)	17 (27)	15 (15)	0.037^a^	19 (22)	13 (18)	0.748^a^
*Chemotherapy*							
Anthracycline + taxane	137 (85)	55 (89)	82 (83)		77 (90)	60 (80)	
Anthracycline	19 (12)	5 (8)	14 (14)		7 (8)	12 (16)	
Taxane	5 (3)	2 (3)	3 (3)	0.508^a^	2 (2)	3 (4)	0.236^a^
*Local or regional tumor recurrence*							
Absent	144 (89)	55 (89)	89 (90)		73 (85)	71 (95)	
Present	17 (11)	7 (11)	10 (10)	0.811^a^	13 (15)	4 (5)	0.044^a^
*Distant metastasis*							
Absent	119 (74)	46 (75)	73 (74)		52 (60)	67 (89)	
Present	42 (26)	16 (25)	26 (26)	0.949^a^	34 (40)	8 (11)	<0.001^a^
*Breast cancer-specific death*							
Absent	127 (79)	47 (76)	80 (81)		57 (66)	70 (93)	
Present	34 (21)	15 (24)	19 (20)	0.449^a^	29 (34)	5 (7)	<0.001^a^

^a^Chi-squared test

### sTILs and TLS in the primary tumor

As per the International Immuno-Oncology Biomarker Working Group guidelines^3^, sTILs were quantified at the primary tumor site and reported as percentage estimates in increments of 10%. The median sTIL level was 10% (standard deviation 17%, range, 0–70%); 47% (75/161) of the carcinomas had ≥20% sTILs. Peritumoral TLS were seen in 24% of cases (38/161) (Table 1, Supplementary Fig. 1), with significantly more frequently in those with ≥20% sTILs than <20% sTILs (32% versus 16%, Chi-squared test, *P* = 0.02, Table 2).

**Table 2:** Germinal centers in involved and cancer-free lymph nodes.

	All cases	HER2	TNBC		sTILs < 20%	sTILs≥20%	
	*n* = 161	*n* = 62	*n* = 99	*P* value	*n* = 86	*n* = 75	*P* value
*LN assessment*							
All LNs, median (range)	17 (10–37)	17 (10–29)	17 (10–37)		17 (10–31)	17 (10–37)	
Cancer-free LNs, median (range)	14 (2–31)	13 (2–24)	16 (3–31)		14 (2–26)	16 (3–31)	
Involved LNs, median (range)	3 (1–18)	4 (1–18)	2 (1–18)		3 (1–17)	3 (1–18)	
GC assessment in LNs per patient basis							
*All LNs, n (%)*							
GC absent	11 (6.8)	5 (8.1)	6 (6.1)		10 (11.6)	1 (1.3)	
GC present	150 (93.2)	57 (91.9)	93 (93.9)	0.624^a^	76 (88.4)	74 (98.7)	0.010^a^
*Cancer-free LNs*							
GC NA^c^	1		1			1	
GC absent	23 (14.4)	10 (16.1)	13 (13.3)		18 (20.9)	5 (6.8)	
GC present	137 (85.6)	52 (83.9)	85 (86.7)	0.615^a^	68 (79.1)	69 (93.2)	0.011^a^
*Involved LNs*							
GC NA^d^	13 (8.1)	4 (6.5)	9 (9.1)		5 (5.8)	8 (10.7)	
GC absent	26 (16.1)	8 (12.9)	18 (18.2)		19 (22.1)	7 (9.3)	
GC present	122 (75.8)	50 (80.6)	72 (72.7)	0.333^a^	62 (72.1)	60 (80)	0.038^a^
*LN number GC present*							
Cancer-free LN, median (range)	3 (1–22)	3 (1–13)	3 (1–22)	0.552^b^	2 (1–17)	4 (1–22)	0.002^b^
Involved LN, median (range)	1 (1–16)	2 (1–16)	1 (1–12)	0.294^b^	1 (1–7)	1 (1–16)	0.598^b^
*Total number of GCs across all assessed LNs per patient*							
Cancer-free LN, median (range)	8 (0–175)	6 (0–142)	9 (0–175)	0.139^b^	6 (0–145)	12 (0–175)	0.002^b^
Involved LN, median (range)	8 (0–214)	9 (0–198)	7 (0–214)	0.508^b^	5 (0–198)	14 (0–214)	0.002^b^
*Max GC number in a LN across all assessed LNs per patient*							
Cancer-free LN, median (range)	5 (0–63)	4 (0–59)	5 (0–63)	0.076^b^	4 (0–59)	6 (0–63)	0.002^b^
Involved LN, median (range)	7 (0–76)	7 (0–76)	6 (0–54)	0.611^b^	3 (0–76)	10 (0–54)	0.003^b^
*Average GC number*							
Cancer-free LN, median (range)	3 (0–35)	3 (0–19)	3 (0–35)	0.091^b^	3 (0–17)	4 (0–35)	0.001^b^
Involved LN, median (range)	5 (0–43)	5 (0–40)	5 (0–43)	0.942^b^	3 (0–40)	8 (0–43)	0.001^b^

^a^Chi-squared test

^b^Mann–Whitney U test

^c^Uninterpretable LN slide

^d^Whole LN involved

### Germinal center formation in cancer-free and involved axillary LNs

A total of 2,212 cancer-free and 645 involved LNs from the 161 breast cancer patients were reviewed; the median was 14 cancer-free LNs (range, 2–31) and 3 involved LNs (range, 1–18) per patient (Table 2). The number of GCs in each LN was assessed and recorded. Cancer-free LNs with more GC numbers showed a weak correlation with larger secondary follicles (Spearman *rho* = *0.29, P* < *0.001*, Supplementary Fig. 2a), and had a predominantly central distribution of the GCs within the LN (peripheral vs predominantly peripheral, Mann–Whitney U test, *P* < *0.001*; peripheral vs predominantly central, Mann–Whitney U test, *P* = *0.001*; Supplementary Fig. 2b). No significant correlation with GC size or significant difference in the distribution of GCs was observed in involved LNs (Supplementary Fig. 2a, b). Across 2,857 LNs, cancer-free and involved LNs with at least 1 GC were found in 137 (86%) and 122 (76%) patients, respectively. Only 7% (11/161) patients had no GCs in any of their nodes (range of assessed LNs per patient, 10–17).

**Fig. 2:**
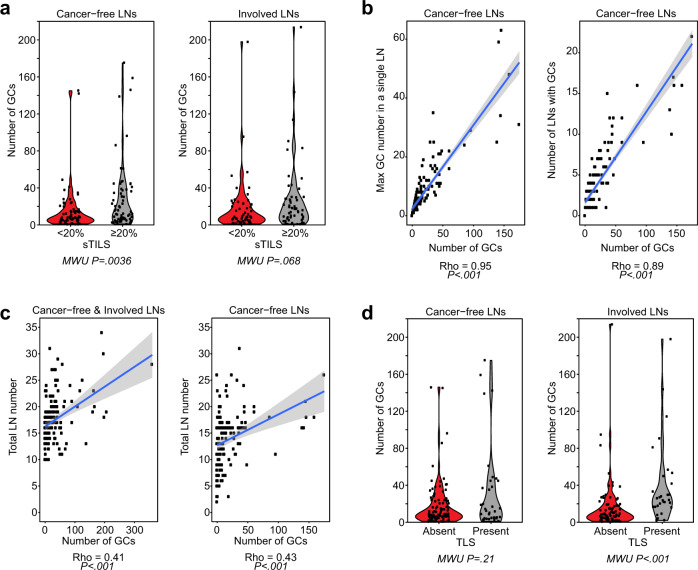
Association between germinal center formation in lymph nodes, stromal TILs and tertiary lymphoid structures. **a** Violin plots, showing the distribution of germinal centers (GCs) compared to sTILs with 20% cut-off (<20% sTILs (*n* = 86) and ≥20% sTILs (*n* = 75)) (X axis), in cancer-free LNs (left), and involved LNs (right). **b** Scatter plot of the number of GCs compared to the maximum number of GCs in a single LN (left side); and compared to the number of LNs which contain GCs (right side). **c** Scatter plots of the number of GCs in all assessed LNs (left) and all cancer-free assessed LNs (right) compared to the number of LNs. **d** Violin plots, showing the distribution of GCs compared to peritumoral TLS (Absent (*n* = 123) and present (*n* = 38)), in cancer-free LNs (left), and involved LNs (right). Mann–Whitney U tests were used to calculate *P* values.

Patients with tumors with fewer sTILs (<20%) at the primary site had more LNs without any GCs (for all LNs, 12% versus 1%, *P* = 0.01; for cancer-free LNs 21% versus 7%, *P* = 0.01; for involved LNs 22% versus 9%, *P* = 0.04, Chi-squared test, Table 2) and fewer total numbers of GC in their cancer-free LNs (Mann–Whitney U test, *P* = *0.036*, Fig. 2a). Considering only patients with any GC formation in their LNs, the median number of cancer-free LNs bearing GCs was statistically higher when sTILs in the primary cancer were ≥20%, compared to those cases where sTILs were <20% (median 4, range, 1–22, versus median 2, range, 1–17, Kruskal–Wallis test, *P* < 0.01, Table 2). No difference in the number of cancer-free LNs with GCs, nor between the number of involved LNs with GCs, was observed between the two breast cancer subtypes (Table 2).

Per patient, the total number of GCs in all of the cancer-free LNs was on average 8 (range, 0–175) and was 8 (range, 0–214) in the total of the involved LNs. In 23/161 (14%) patients ALNC was performed after positive sentinel lymph node biopsies, allowing the comparison of GC formation in sentinel versus other axillary LNs (Supplementary Table 1). In patients with >2 GCs in all assessed cancer-free LNs, the majority of GCs were observed in LNs excised by SLNBs, including involved and cancer-free nodes, in comparison to nodes obtained by ALNC. In 4/23 patients with SLNB (#20, #21, #22 and #23), neither cancer-free nor involved LNs displayed any GC formation. In patient #19, where a total of 2 GCs were observed amongst all assessed cancer-free LNs, a single GC formation was observed in a node excised by SLNB, whilst the other was in an axillary LN.

When the number of GCs was compared in individual cancer-free and involved LNs, this harbored a median of 3 (range, 0–35) and 5 (range, 0–43), respectively (Table 2). In the group of carcinomas with ≥20% sTILs: (i) the total GC numbers were higher in both cancer-free and involved LNs compared to those with <20% sTILs; (ii) the maximum GC number in a cancer-free and involved LNs was greater; and (iii) on average any one individual cancer-free or involved LN had more GCs (Table 2). Furthermore, the total number of GCs per patient correlated with the maximum GC number (Spearman *rho* = *0.95, P* < *0.001*, Fig. 2b; Supplementary Fig. 2c) and with the number of LNs with GCs in cancer-free LNs (Spearman *rho* = *0.89, P* < *0.001*, Fig. 2b; Supplementary Fig. 2c). However, only a moderate correlation was observed between the total number of GCs and the number of assessed LNs, when including both cancer-free and involved LNs (Spearman *rho* = *0.41, P* < *0.001*, Fig. 2c; Supplementary Fig. 2d), and when only cancer-free assessed LNs were tested (Spearman *rho* = *0.43, P* < *0.001*, Fig. 2c, Supplementary Fig. 2d). Given, the correlation amongst these different GC assessments, and their independence to the number of assessed LNs, the total number of GCs per patient was used for further analyses.

### Association of GC numbers in LNs with clinicopathological features

Patients with TLS adjacent to the primary carcinomas had more GCs in their involved LNs, but not in their cancer-free LNs (Mann–Whitney U test, *P* < 0.001 and *P* = 0.21, respectively, Fig. 2d). The number of GCs in the total cancer-free LNs per patient decreased slightly with age at diagnosis (Spearman *rho* = *−*0.32, *P* < 0.001, Supplementary Fig. 2e). The GC number in involved LNs increased with nodal status (Mann–Whitney U test, *P* = 0.02, Supplementary Fig. 2f). No association was observed between GC number (either in involved or cancer-free LNs) with tumor size or the presence of lympho-vascular invasion (Mann–Whitney U test, *P* > 0.05; Supplementary Fig. 2g, h).

### Association of GC number in LNs with prognosis

**Fig. 3:**
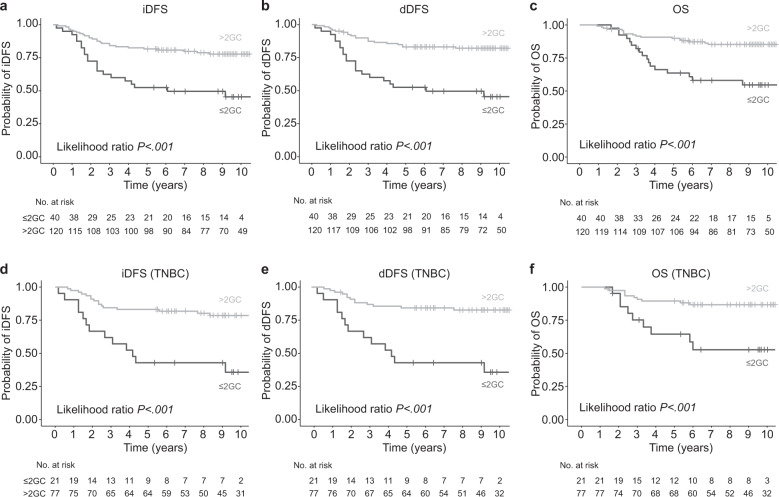
Kaplan-Meier survival analyses predicting. **a** invasive Disease-Free Survival (iDFS), **b** distant Disease-Free Survival (dDFS), **c** Overall Survival (OS), **d** invasive Disease-Free Survival (iDFS) in TNBC, **e** distant Disease-Free Survival (dDFS) in TNBC and **f** Overall Survival (OS) in TNBC. Patients were dichotomized into those with ≤ 2GCs versus >2 GCs in all assessed cancer-free LNs. *P* values correspond to likelihood ratio tests.

In concordance with recent research^4^, an increased sTILs density was associated with improved outcome for all endpoints (invasive Disease Free Survival (iDFS): hazard ratio (HR) = 0.96, 95% confidence interval (CI) 0.93–0.98, *P* < 0.001; dDFS: HR = 0.96, 95%CI 0.93–0.98, *P* < 0.001; overall survival (OS): HR = 0.94, 95%CI 0.91–0.98, *P* < 0.001; Supplementary Table 2). The presence of TLS was also associated with an improved outcome for all endpoints (iDFS: HR = 0.25; 95% CI 0.09–0.71, *P* < 0.001; dDFS: HR = 0.21, 95% CI 0.06–0.67, *P* = 0.001; OS: HR = 0.08, 95% CI 0.01–0.59, *P* < 0.001; Supplementary Table 2). To consolidate whether the number of GCs across all assessed cancer-free LNs is associated with prognosis in this cohort, as we have shown previously^11^, we performed an iterative process to determine an optimal cut-off point by a minimal *P* value approach^12^ (Supplementary Fig. 3), which revealed that patients with ≤2 GCs across all assessed cancer-free LNs had poorer iDFS, dDFS and OS than patients with >2 GCs in all assessed cancer-free LNs (Table 3, Fig. 3a–c). In multivariate models, when adjusted for known prognostic factors and TILs, this binary cut-off for GCs in cancer-free LNs remained statistically associated with dDFS (HR = 0.47, 95% CI 0.23–0.94, *P* = 0.033; Table 3), and increased in significance when only TNBC patients (*n* = 99) were analyzed (iDFS: HR = 0.37; 95% CI 0.16–0.84, *P* = 0.017; dDFS: HR = 0.29, 95% CI 0.13–0.67, *P* = 0.004; Supplementary Table 3, Fig. 3d-f). In the subset of HER2-positive patients (*n* = 62), those patients with >2 GC in all assessed cancer-free LNs had better OS (OS: HR = 0.33, 95% CI 0.12–0.92, *P* = 0.036; Supplementary Table 2); however, these significant associations were lost in the multivariate analyses (Supplementary Table 3).

**Table 3:** Univariate and multivariate Cox regression analyses of germinal center numbers in cancer-free LNs for iDFS, dDFS, and OS of HR-negative, their TILs subgroups, all TNBC and low TILs TNBC.

	Covariate P	Model P	HR	CI
(A) All HR-negative cases				
iDFS				
All cases (*n* = 161)				
Univariate - Total GCs number (≤2 *v* > 2)	/	<0.001	0.33	0.19–0.59
Multivariate^a^ - Total GCs number (≤2 *v* > 2)	0.110	<0.001	0.58	0.30–1.12
<20% sTILs (*n* = 86)				
Univariate - Total GCs number (≤2 *v* > 2)	/	0.002	0.36	0.19–0.69
Multivariate^b^ - Total GCs number (≤2 *v* > 2)	0.023	0.004	0.41	0.19–0.89
≥20% sTILs (*n* = 75)				
Univariate - Total GCs number (≤2 *v* > 2)	/	0.804	1.29	0.16–10.16
Multivariate^b^ - Total GCs number (≤2 *v* > 2)	0.949	0.034	0.93	0.11–7.93
dDFS				
All cases (*n* = 161)				
Univariate - Total GCs number (≤2 *v* > 2)	/	<0.001	0.26	0.14–0.48
Multivariate^a^ - Total GCs number (≤2 *v* > 2)	0.033	<0.001	0.47	0.23–0.94
<20% sTILs (*n* = 86)				
Univariate - Total GCs number (≤2 *v* > 2)	/	<0.001	0.28	0.14–0.56
Multivariate^b^ - Total GCs number (≤2 *v* > 2)	0.009	<0.001	0.34	0.17–0.77
≥20% sTILs (*n* = 75)				
Univariate - Total GCs number (≤2 *v* > 2)	/	0.986	1.02	0.13–8.29
Multivariate^b^ - Total GCs number (≤2 *v* > 2)	0.665	0.031	0.61	0.07–5.64
OS				
All cases (*n* = 161)				
Univariate - Total GCs number (≤2 *v* > 2)	/	<0.001	0.28	0.14–0.55
Multivariate^a^ - Total GCs number (≤2 *v* > 2)	0.351	<0.001	0.69	0.32–1.50
<20% sTILs (*n* = 86)				
Univariate - Total GCs number (≤2 *v* > 2)	/	0.006	0.36	0.17–0.75
Multivariate^b^ - Total GCs number (≤2 *v* > 2)	0.106	0.001	0.48	0.20–1.17
≥20% sTILs (*n* = 75)				
Univariate - Total GCs number (≤2 *v* > 2)	/	0.652	0.59	0.07–5.25
Multivariate^b^ - Total GCs number (≤2 *v* > 2)	-^c^	-^c^	-^c^	-^c^
(B) Triple-negative breast cancers				
iDFS				
All cases (*n* = 99)				
Univariate - Total GCs number (≤2 *v* > 2)	/	<0.001	0.25	0.12–0.52
Multivariate^a^ - Total GCs number (≤2 *v* > 2)	0.017	<0.001	0.37	0.16–0.84
<20% sTILs (*n* = 52)				
Univariate - Total GCs number (≤2 *v* > 2)	/	<0.001	0.25	0.11–0.57
Multivariate^b^ - Total GCs number (≤2 *v* > 2)	0.003	0.016	0.26	0.10–0.64
dDFS				
All cases (*n* = 99)				
Univariate - Total GCs number (≤2 *v* > 2)	/	<0.001	0.20	0.09–0.44
Multivariate^a^ - Total GCs number (≤2 *v* > 2)	0.004	<0.001	0.29	0.13–0.67
<20% sTILs (*n* = 52)				
Univariate - Total GCs number (≤2 *v* > 2)	/	<0.001	0.21	0.09–0.49
Multivariate^b^ - Total GCs number (≤2 *v* > 2)	0.001	0.004	0.21	0.08–0.55
OS				
All cases (*n* = 99)				
Univariate - Total GCs number (≤2 *v* > 2)	/	0.004	0.24	0.10–0.60
Multivariate^a^ - Total GCs number (≤2 *v* > 2)	0.119	<0.001	0.46	0.17–1.22
<20% sTILs (*n* = 52)				
Univariate - Total GCs number (≤2 *v* > 2)	/	0.013	0.29	0.11–0.76
Multivariate^b^ - Total GCs number (≤2 *v* > 2)	0.036	0.005	0.32	0.11–0.93

^a^Adjusted for Age, pTstage, pNstage, LVI, sTILs & TLS

^b^Adjusted for Age, pTstage, pNstage, LVI & TLS

^c^Group size too small/too few events.

**Fig. 4:**
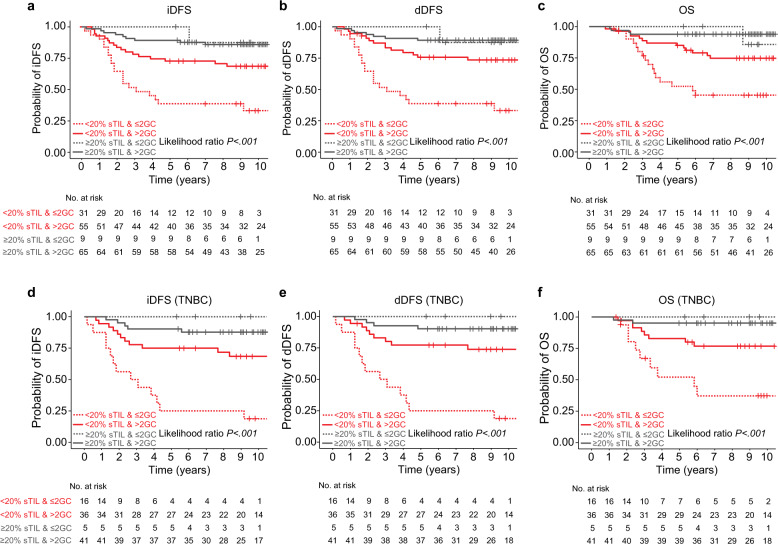
Association between germinal center formation in lymph nodes and prognosis in HR-negative breast cancers. Kaplan–Meier curves: **a** invasive Disease-Free Survival (iDFS), **b** distant Disease-Free Survival (dDFS), **c** Overall Survival (OS), **d** invasive Disease-Free Survival (iDFS) in TNBC, **e** distant Disease-Free Survival (dDFS) in TNBC, and **f** Overall Survival (OS) in TNBC, according to stromal tumor-infiltrating lymphocytes (TILs) and germinal center (GC) number. Patient groups were stratified by TILs (≥20%, <20%) and the number of GCs (≤ 2GCs, > 2GC) in all assessed cancer-free LNs, as categorical variables. *P* values correspond to likelihood ratio tests.

Next, we asked whether the positive prognostic effect of the systemic immune response in cancer-free LNs differs in patients with different sTILs at the primary lesion. In patients with high sTILs tumors, the frequency of GCs in cancer-free LNs had no influence on disease trajectories. However, in univariate and multivariate models, patients with low sTIL tumors and >2 GCs in cancer-free LNs in comparison to those with ≤2 GC frequency had superior dDFS (HR = 0.34, 95% CI 0.17–0.77, *P* = 0.009), and iDFS (HR = 0.41, 95% CI 0.19–0.89, *P* = 0.023), and a tendency in OS (HR = 0.48, 95% CI 0.2–1.17, *P* = 0.106; Fig. 4a–c and Table 3a). Subgroup analyses demonstrated that this association was driven by the subset of TNBC (*n* = 99), in which patients >2 GC in all assessed cancer-free LNs had better dDFS (HR = 0.21, 95% CI 0.08–0.55, *P* = 0.001), iDFS (HR = 0.26, 95% CI 0.1–0.64, *P* = 0.003), and OS (HR = 0.32, 95% CI 0.11–0.93, *P* = 0.036; Fig. 4d–f and Table 3b).

The five-year iDFS, dDFS and OS in patients with <20% sTILs was 39%, 39% and 52% respectively for those with ≤2 GCs whilst those with >2 GCs had five-year iDFS, dDFS and OS of 73%, 76% and 85%, respectively. As 66/75 (88%) patients with high sTILs tumors have >2 GC in cancer-free LNs, the five-year iDFS, dDFS and OS could only be estimated in this subgroup and was 89%, 89%, and 94%, respectively (Table 4a). In the subset of TNBC with <20% sTILs, patients with ≤2 GCs in their cancer-free LNs had five-year iDFS, dDFS and OS of 25%, 25%, and 52% respectively, in comparison to patients with >2 GCs in their cancer-free LNs who had five-year iDFS, dDFS and OS of 75%, 77%, and 82%, respectively (Table 4b), illustrating a prognostic value for the number of GC formation in low TILs TNBCs.

**Table 4:** 5-year outcome for patients by TILs in primary cancers & germinal center subgroups. A) All HR-negative cases. B) Triple-negative breast cancers.

	Number (%)	5-Year iDFS (95% CI)	5-Year dDFS (95% CI)	5-Year OS (95% CI)
(A) All HR-negative cases				
*Low sTILs (n* *=* *86)*				
≤2 GCs	31 (36)	39 (22–55)	39 (22–55)	52 (33–68)
>2 GCs	55 (64)	73 (59–83)	76 (62–85)	85 (72–92)
*High sTILs (n* *=* *75)*				
≤2 GCs	9 (12)	100 (−)	100 (−)	100 (−)
>2 GCs	66 (88)	89 (79–95)	89 (79–95)	94 (84–98)
(B) Triple-negative breast cancers				
*Low sTILs (n* *=* *52)*				
≤2 GCs	16 (31)	25 (8–47)	25 (8–47)	52 (25–74)
>2 GCs	36 (69)	75 (58–86)	77 (60–88)	82 (66–92)
*High sTILs (n* *=* *47)*				
≤2 GCs	5 (11)	100 (−)	100 (−)	100 (−)
>2 GCs	42 (89)	90 (76–96)	90 (76–96)	95 (82–99)

## Discussion

We describe here, in TNBC and HER2-positive cancer patients, the largest set to date of cancer-free and involved axillary LNs with matched primary tumors and show that humoral, systemic immune responses at the time of primary surgery have prognostic value. Thus, this study supports and extends our previous findings^11^, since particularly in TNBC patients with low sTIL tumors, time to progression of disease was prolonged when their LNs displayed some indications of immune response. The better outcome in patients with GC formation in their cancer-free LNs, even when stromal TILs are low in the primary lesion, alludes to a systemic anticancer immune response. This data indicates that pathological assessment of GCs in cancer-free LNs, in conjunction with TILs, is of value for prognostication in high-risk patients.

All patients in this series had primary therapeutic breast surgery and axillary LN clearance, so that any anti-tumor immune response beyond that at the primary tumor site could be examined. Other models have already highlighted the importance of this systemic response; for example, successful tumor eradication after immunotherapy in genetically engineered cancer models required immune activation in the periphery^13^; and recently, Hollern and colleagues have elegantly illustrated how T follicular helper (Tfh) cell activation of B cells can facilitate anti-tumor responses to immune checkpoint inhibitors^14^. A productive GC response requires the collaboration of multiple cell types. Although the underlying stimuli that results in GC formation in breast cancer are incompletely understood, after infection or vaccination, GCs are transiently formed as B cell follicles of secondary lymphoid tissues^15^ with clonal expansion of B cells, ensuring the development of long-lived pathogen-specific humoral immunity.

We observed an inverse relationship between the number of GCs in LNs and the age of the patient at diagnosis, which is in alignment with a decreased GC prevalence and volume in LNs in elderly patients, potentially resulting in a decrease in LN’s reactivity^16^. While B cells still retain the ability to migrate in aging LNs and produce immunoglobulin, the number of follicular dendritic cells in LNs and the ability to hold on to immune complexes is significantly impaired, potentially as a result of poor humoral immunity in the older patients^17^. In alignment with previous reports, patients with high sTILs in the primary tumor had not only more TLS but also more GCs^18,19^. Both of these lymphoid structures may potentially indicate an effective humoral immune response in these patients, who, in general, have a better prognosis. Deciphering the fundamental drivers of GC formation in LNs in breast cancer patients may reveal mechanisms underpinning the generation of robust humoral immunity and thus identify strategies to potentially target the modulation of GCs in cancer.

Increased pathological complete response is reported in clinical trials of TNBC patients when immune checkpoint blockade immunotherapies (e.g. anti-PD1/PDL1) are combined with chemotherapy^20,21^, and in patients with high sTILs^6^. In particular, LN-positive patients showed a greater benefit to immune checkpoint inhibitors with neoadjuvant chemotherapy in the randomized Phase III KEYNOTE-522 trial, than patients with lower risks (∆21% for node-positive and ∆25% for stage IIIA/B disease breast cancer patients)^22^. We postulate that the systemic immune responses in node-positive breast cancer patients may be advantageous for immune checkpoint inhibitors therapy response. By further exploring these systemic immune responses (i.e. in LNs), we will expand on our understanding of why some patients are more likely respond to these immunotherapies.

In the present study, a significant survival improvement for LN-positive patients with low TILs was observed when cancer-free LNs harbored >2 GCs for all patient outcomes examined. In particular, the presence of numerous GCs may indicate immune responses in a patient that are not captured by their sTILs levels at the primary tumor site at the time when the tumor is histopathologically assessed. We cannot comment on whether immune responses were previously present, however the reactivity of these secondary follicles indicates the patient’s ability to mount an immune response, and potentially represents a component contributing to the better disease trajectory for these patients compared to patients without any local and systemic immune responses (*i.e*. with both low sTILs & low GC numbers). A functional influence on lymphocytes at the primary cancer by immune checkpoints in LNs has already been proposed^19^, also corroborating a close connection between the primary tumor and adjacent LNs.

Of note 38% patients in the present study had HER2-positive tumors, and it is possible that an assessment of systemic immune response by examination of GCs in addition to TILs may be of predictive importance for these patients; in the A TRYPHAENA substudy those with low TILs had an inferior response to trastuzumab/pertuzumab-based chemotherapy^5^. However, our study was not intended to analyze interactions with chemotherapy or targeted agents and further research is needed to determine whether the assessment of GCs in cancer-free LNs provides additive value for prediction of immunotherapy or anti-HER2 treatment response. Recent studies have brought attention to the role of B cells, especially within TLS, which act akin to LNs within a tumor, and have noted that B cell presence is critical for response to checkpoint blockade, thereby pointing to a dynamic interaction between several components of the immune system^23^. Thus, understanding the bipartite nature of the immune system may then help to identify patient subgroups for whom targeting both T cells and B cells could improve treatment response.

Given the retrospective nature of this study, further analytical and clinical validation, as well as evaluation of reproducibility of assessment of GCs, is required. Ideally, this would be undertaken on samples from patients in clinical trials, with uniform management and follow-up, but the LNs (involved or cancer-free) from such women are not typically curated in clinical trials tissue banks; this should be considered in future. Assessment of the LNs from patients within neoadjuvant chemotherapy trials for GC numbers would provide evidence of value in this setting. Indeed, TILs have been examined in this setting and residual cancer burden (RCB) is used as an endpoint^24^, thus and this approach would similarly provide an excellent opportunity to consolidate our results.

In 14% of our study cohort, SLNB was performed, suggesting that capturing data on GC formation in SLN can reflect on the frequency of GC formation overall in axillary LNs in these patients. However, further studies are warranted to evaluate the minimum number of nodes required and whether the cut-point for GC numbers are the same. The proposed cut-offs for GC numbers in cancer-free LNs may also then need revision. Conversely, the examination and counting of GCs in all LNs in an axillary clearance requires additional pathology time and resources. Convolutional neural networks applied to digitized whole slide images can detect LN metastasis with high accuracy in some studies^25^ and digital pathological approaches to the quantification of TILs have also been described^26^. The histology of GCs is suited to be captured by machine learning methods^27^ and will potentially facilitate assessment in large cohorts and additional numbers of cases of all breast cancer subtypes.

In conclusion, we show that systemic immune response at the time of primary surgery, by the recording of GC formation in the cancer-free LNs, has prognostic value. This highlights that axillary LN assessment, above and beyond the presence and size of cancer cell deposits, in conjunction with sTILs, carries prognostic value in high-risk patients.

## Methods

### Patients

Patient selection and data analyses are reported according to Reporting Recommendations for Tumor Marker Prognostic Studies (REMARK) criteria^28^. Ethical clearance was obtained from the local research ethics committee (Medical Ethics Committee of Tianjin Medical University Cancer Institute and Hospital, Ek2020021). This is a retrospective study of 161 patients with invasive breast carcinoma of no special type (IBC-NST) treated between 2005 and 2010 at Tianjin Medical University Cancer Hospital, China, consisting of HR-negative patients (HER2-positive or TNBC) with positive LNs and of histological grade 3 (Fig. 1). The median age at diagnosis was 52 years (range, 23–75). All patients underwent modified radical mastectomy or breast-conserving surgery and had axillary LN dissection. None of the patients had prior history of breast or axillary surgery, or suffered from Small Lymphocytic Lymphoma, Chronic Lymphocytic Leukemia, dermatopathic lymphadenopathy, benign inflammatory disease of the breast or upper limb. None had neoadjuvant systemic therapy. Postoperatively, all patients received adjuvant chemotherapy; 85% anthracycline plus taxane, 12% anthracycline-based (and another 3% taxane only-based chemotherapy (Table 1). In this period HER2-positive patients in China did not receive any anti-HER2 therapy.

Clinicopathological data are recorded in Table 1.

### Histopathological assessment of primary tumor and LNs

Routine H&E-stained sections of formalin-fixed paraffin embedded tissue from the primary invasive breast carcinoma and involved and cancer-free LNs were scanned at ×40 magnification using a NanoZoomer HT Digital Pathology Scanning System (Hamamatsu, Japan). All sections were reviewed by two breast pathologists (FL and XG) who assessed the presence and number of GCs, TILs and TLSs. A total of 2857 axillary LNs from 161 patients were obtained, with an average of 5 sections per primary tumor and 10 to 37 (median, 17) LNs per patient.

As per the International Immuno-Oncology Biomarker Working Group guidelines^3^, sTIL density was quantitatively assessed and reported as a percentage estimate, in increments of 10%. Patient groups were dichotomized into those with <20% or ≥20% sTIL, in keeping with recent literature^24,29^. TLS were defined as a follicular structure in the peritumoral stroma on H&E stains^30^, and were reported as present or absent (Supplementary Fig. 1). No immunohistochemical stains for immune cells were used, so this may represent an underestimation of TLS numbers, but represents day-to-day pathology practice. Under conditions of antigenic stimulation, LNs develop secondary follicles composed of a peripheral area of closely packed, small lymphocytes and a centrally located GC. We defined GCs in H&E-stained sections as lighter areas within the small mature lymphoid population composed of both larger lymphoid cells and cells of a non-lymphoid nature. The pathologist chose one of the LN slices with the most GCs and recorded the number of GCs in one LN. Using the NDP.view software of the NanoZoomer Scanning System, the size of each GC, defined as the maximum dimension, was recorded as a continuous variable. The localization of GCs within LNs was classified as peripheral, predominantly peripheral (more GCs close to the capsule), central and predominantly central (more GCs in the center of the LN), as previously described^11^.

### Statistical analysis

Standard summary statistics were performed, to establish if there were associations between GC number, sTILs, TLS and clinicopathological characteristics and with patient outcome. The primary endpoint was distant Disease Free Survival, defined as the date of first distant recurrence or death from any cause. Invasive Disease Free Survival was defined as the date of first invasive recurrence, or second primary, or death from any cause^31^. Overall Survival was defined as the date of death from any cause. For all these analyses patients still alive were censored at the date of the last visit.

A Kaplan-Meier method was used to visualize survival curves and the log-likelihood test to compare survival curves across groups. Follow-up was curtailed at 10 years because of the declining numbers of patients after this time point. Cox regression proportional hazards models were performed to estimate the hazard ratios according to clinicopathological and histological-assessed features across all endpoints in univariate and multivariate analyses. Statistical significance of features was assessed using the log-likelihood test whereby a two-sided *P* < 0.05 was considered significant. Statistical analyses were performed in the statistical environment R 3.5.1.

### Reporting summary

Further information on research design is available in the Nature Research Reporting Summary linked to this article.

## Supplementary information

Supplementary Data 1

Supplementary Information

Reporting Summary

## Data Availability

The data generated and analyzed during this study are described in the following data record: 10.6084/m9.figshare.14589063^32^. All data are openly available together with the data record in the file ‘LymphNodeMorphologicalAssessment_Liu.txt’. The file contains count data for the assessment of morphological features of cancer-free and involved lymph nodes of hormone-receptor negative breast cancers. In addition, it lists TILs scores and detailed clinicopathological data.

## Code availability

Available upon request.
