# Supplementary material for: Systemic immune reaction in axillary lymph nodes adds to tumor-infiltrating lymphocytes in triple-negative breast cancer prognostication
Source: NPJ Breast Cancer. 2021 Jul 5;7:86. doi: 10.1038/s41523-021-00292-y (PMC8257702; doi:10.1038/s41523-021-00292-y)
Supplement: Supplementary file 2 — Supplementary Information [file 41523_2021_292_MOESM2_ESM.pdf]

Data Supplement

Supplementary Figure 1.

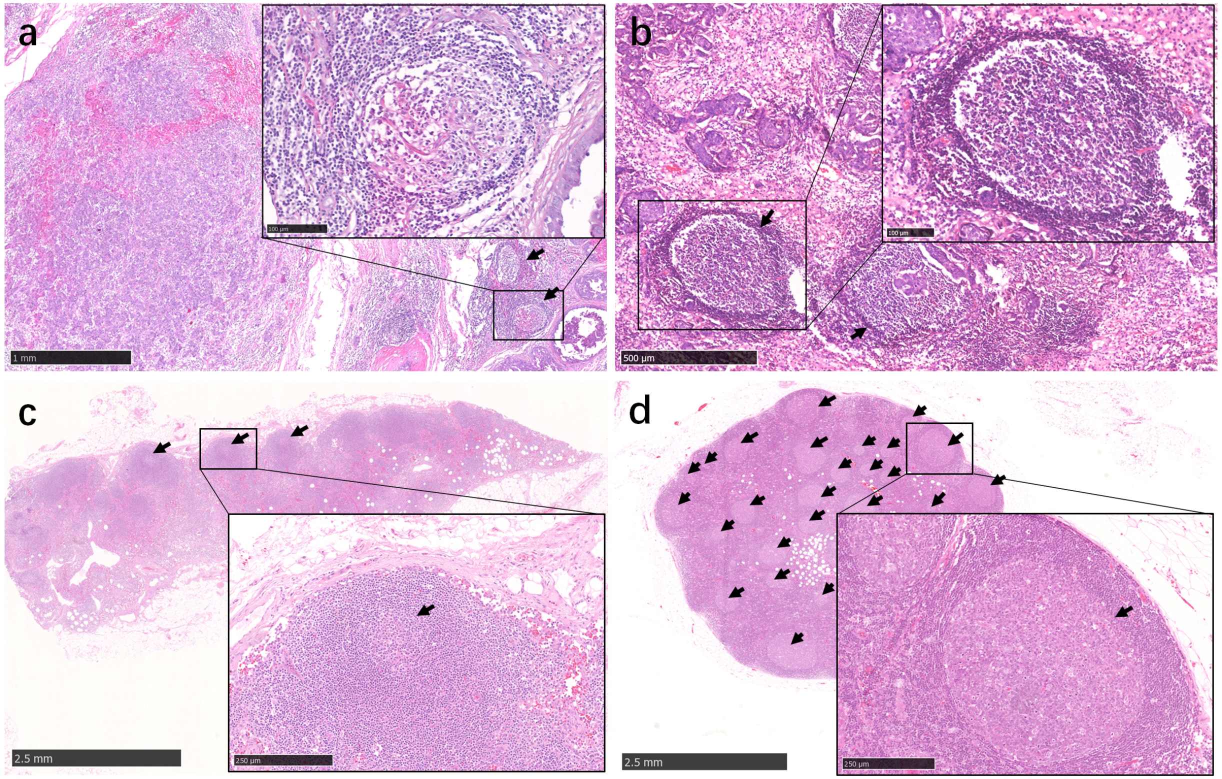

Supplementary Figure 1. Examples of tertiary lymphoid structures (TLS) in primary tumor and germinal centers (GCs) in cancer-free lymph nodes. (a, b) presence of TLS (with lymphoid GC formation, arrows) in the peritumoral stroma (inset showing a higher power view of TLS); **(c)** few GCs (arrow) located close to the LN capsule (predominantly in periphery) (inset showing a higher power view of GC); **(d)** numerous GC formation (arrow); larger, rounder and fuller GCs throughout the whole LN (predominantly in the central part of the LN) (inset showing a higher power view of GC).

Supplementary Figure 2.

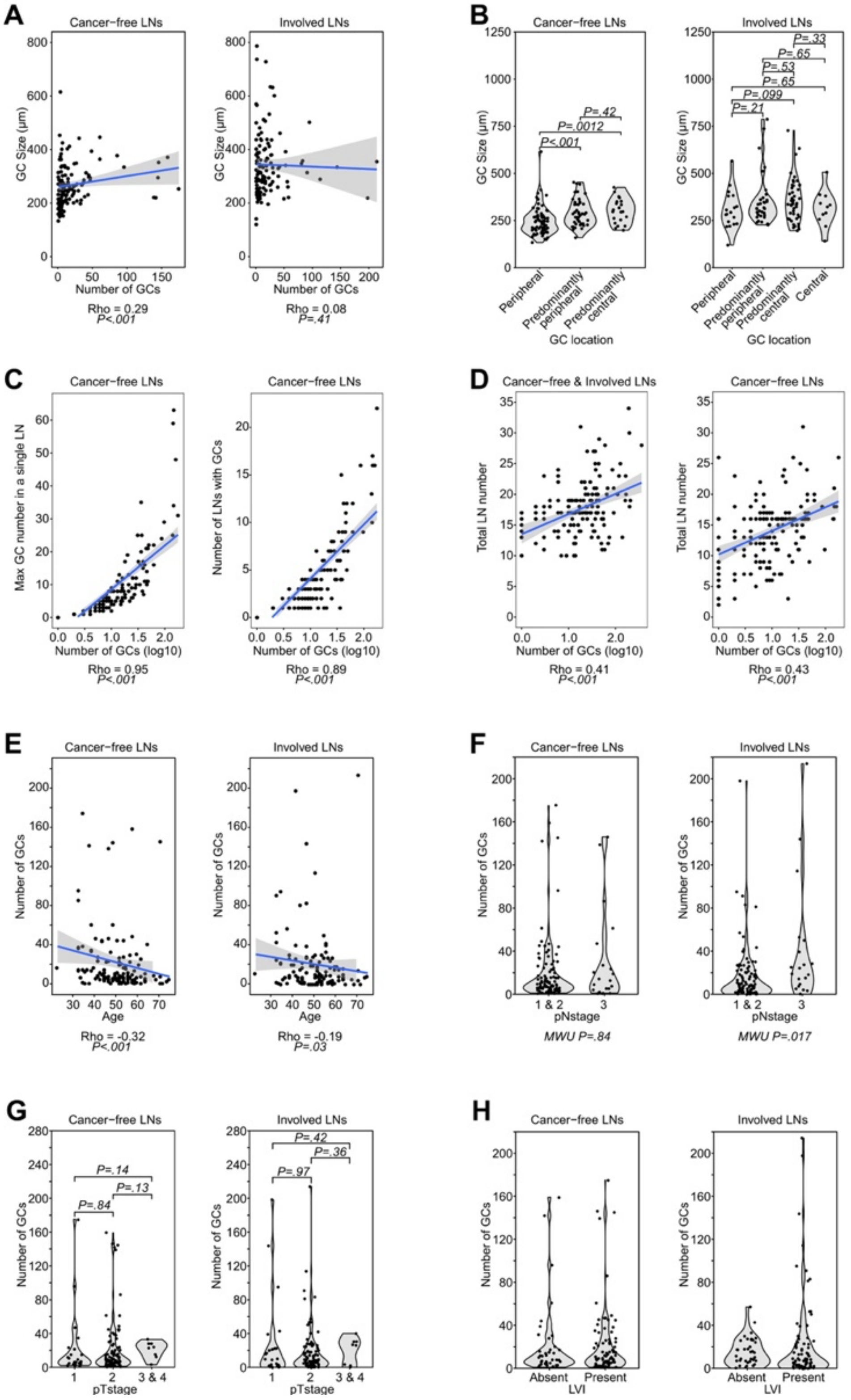

Supplementary Figure 2. Association of the number of germinal centers in cancer-free and involved lymph nodes with clinicopathological features.

(**a**) Scatter plots of the number of GCs compared to the maximum GC size. Cancer-free and involved LNs analysis is shown on the left and right, respectively. (**b**) Violin plot of maximum GC size in GC with regards to their predominant location in the LN (Peripheral (cancer-free n = 70 & involved n= 18), predominantly peripheral (cancer-free n = 70 & involved n = 34), predominantly central (cancer-free n = 21 & involved n= 11) and central (cancer-free n = 0 & involved n= 21)). P values displayed calculated via Mann-Whitney U tests. Cancer-free and involved LNs analysis is shown on the left and right, respectively. (**c**) Scatter plot of the log10 number of GCs compared to the maximum number of GCs in a single LN (left side); and compared to the number of LNs which contain GCs (right side). (**d**) Scatter plots of the log10 number of GCs in all assessed LNs (left) or cancer-free LNs (right) compared to the number of LNs (including those with zero GCs). (**e**) Scatter plots of the number of GCs in cancer-free LNs (left) or involved LNs (right) compared to age at diagnosis; (**f**) Violin plot of the number of GCs in cancer-free LNs (left) or involved LNs (right) involved LNs (Y axis) compared to LN stage (pNstage 1&2 (n = 129) and pNstage 3 (n = 32)) ; (**g**) Violin plot, showing the distribution of GC formations in cancer-free LNs (left) or involved LNs (right) compared to the size of the primary tumor (pTstage 1 (n = 33), pNstage 2 (n = 116) and pNstage 3 & 4 (n = 12)). P values displayed calculated via Mann-Whitney U test; (**h**) Violin plots, displaying the number of GCs in cancer-free LNs (left) or involved LNs (right) compared to patients with (n = 51) and without (n = 110) lympho-vascular invasion.

Supplementary Figure 3.

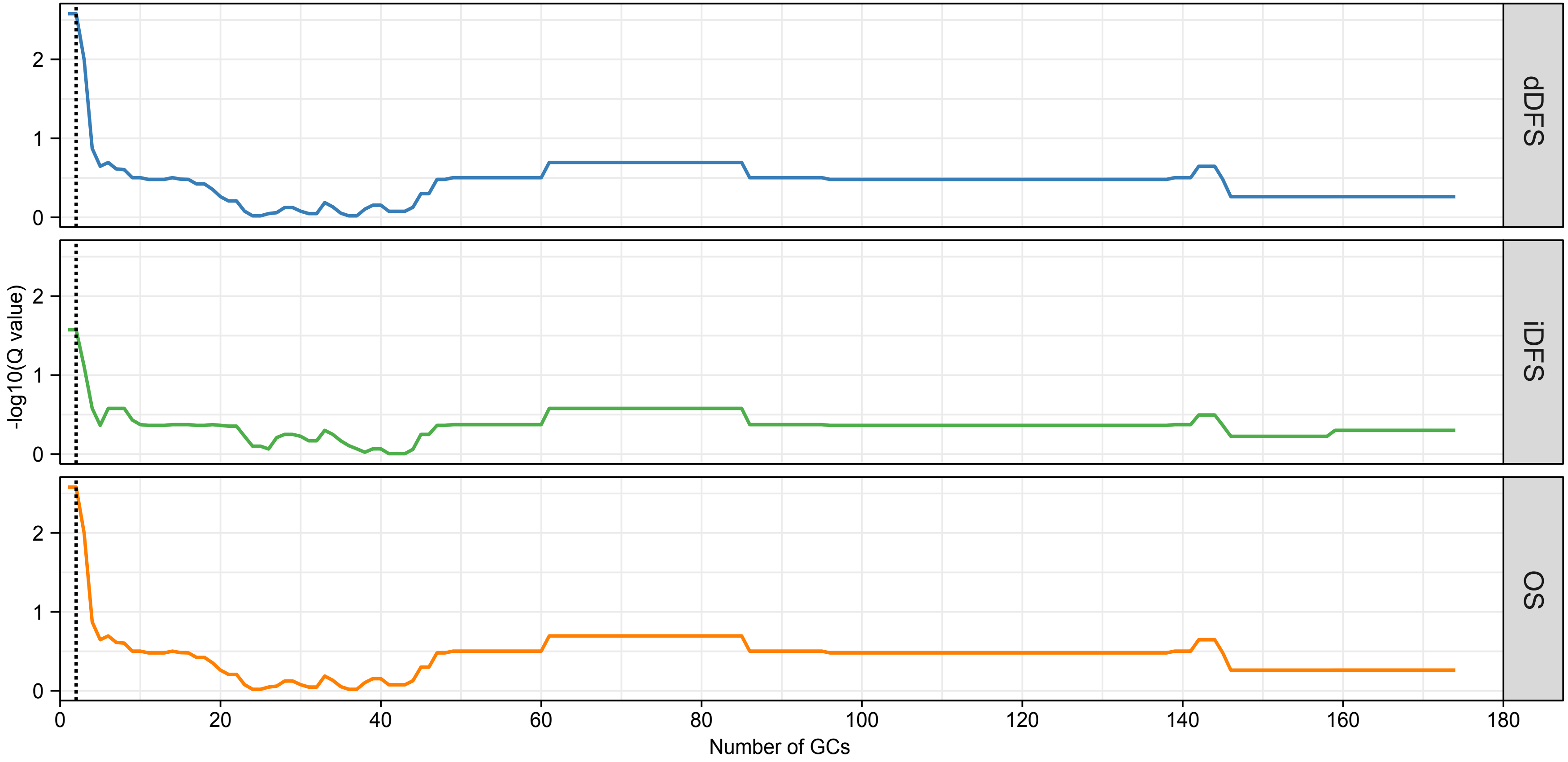

Supplementary Figure 3. Cut off selection for defining the number of germinal centers in cancer-free lymph nodes. The optimal germinal center number cut off was determined by taking all patients (including those with zero GCs) and applying an iterative process using a minimal p value approach. For all three endpoints a single cut-off was identified (dashed black lines) at 2 germinal centers.

Supplementary Table 1. The distribution of total GCs in LNs excised by SLNB and ALNC. Patients are listed who had a SLNB followed by an ALNC. For each patient, the numbers of LNs and numbers of GCs in cancer-free and involved LNs are listed separately. Within cancer-free and involved LNs, the number of LNs excised by SLNB and by ALNC (called non-SLN) are shown. The number of GCs in SLN and non-SLN, as well as the percentage of GC formation given the total number of GCs observed in all cancer-free or involved LNs (referred to as % of total) are reported. The median number of LNs, GCs and percentage of GC formation is shown.

Patient ID		Cancer-free LNs										Involved LNs							
		Total LNs		SLN			non-SLN			Total LNs		SLN			non-SLN		
		Number of LNs	Number of GCs	Number of LNs	GCs		Number of LNs	GCs		Number of LNs	Number of GCs	Number of LNs	GCs		Number of LNs	GCs	
					Number	% of total		Number	% of total				Number	% of total		Number	% of total
case 1	83787	16	142	2	101	71%	14	41	29%	2	57	2	57	N/A	0	N/A	
case 2	84658	26	24	2	12	50%	24	12	50%	1	12	1	12	N/A	0	N/A	
case 3	93074	16	7	2	6	86%	14	1	14%	1	2	1	2	N/A	0	N/A	
case 4	90419	18	159	5	124	78%	13	35	22%	1	2	1	2	N/A	0	N/A	
case 5	90507	16	6	1	6	100%	15	0	0%	1	0	1	0	N/A	0	N/A	
case 6	91212	18	6	2	5	83%	16	1	17%	1	7	1	7	N/A	0	N/A	
case 7	92028	11	12	2	8	67%	9	4	33%	2	30	2	30	N/A	0	N/A	
case 8	94096	13	7	3	4	57%	10	3	43%	2	8	2	8	N/A	0	N/A	
case 9	96285	9	5	2	4	80%	7	1	20%	3	7	3	7	N/A	0	N/A	
case 10	100631	9	6	1	5	83%	8	1	17%	1	0	1	0	N/A	0	N/A	
case 11	101812	20	61	5	47	77%	15	14	23%	1	30	1	30	N/A	0	N/A	
case 12	103272	20	45	3	29	64%	17	16	36%	6	81	3	61	75%	3	20	25%
case 13	105096	18	34	3	24	71%	15	10	29%	2	7	2	7	N/A	0	N/A	
case 14	108069	13	38	3	23	61%	10	15	39%	3	53	1	42	79%	2	11	21%
case 15	108630	20	4	2	2	50%	18	2	50%	2	17	1	14	82%	1	3	18%
case 16	108631	17	7	1	6	86%	16	1	14%	1	9	1	9	N/A	0	N/A	
case 17	103813	24	16	3	11	69%	21	5	31%	1	27	1	27	N/A	0	N/A	
case 18	107221	11	6	3	4	67%	8	2	33%	1	6	1	6	N/A	0	N/A	
case 19	81543	16	2	1	1	50%	15	1	50%	2	0	1	0	N/A	1	0	N/A
case 20	93805	11	0	1	0	N/A	10	0	N/A	2	0	1	0	N/A	1	0	N/A
case 21	108731	16	0	2	0	N/A	14	0	N/A	1	0	1	0	N/A	0	N/A	
case 22	101463	9	0	1	0	N/A	8	0	N/A	1	0	1	0	N/A	0	N/A	
case 23	106265	9	0	1	0	N/A	8	0	N/A	1	0	1	0	N/A	0	N/A	
**Median**		16	7	2	6	71%	14	2	29%	1	7	1	7	79%	0	3	21%

Supplementary Table 2. Univariate & Multivariate Cox regression for iDFS, dDFS and OS of all cases or sTILs subgroups (< 20%, ≥ 20%)

Supplementary Table 2a. Univariate and Multivariate Cox regression analyses for iDFS

iDFS

Univariate	All cases			<20% sTILs			≥20% sTILs		
**Clinicopathological features**	**Model P**	**HR**	**CI**	**Model P**	**HR**	**CI**	**Model P**	**HR**	**CI**
**Age at diagnosis**	0.123	1.02	0.99 - 1.05	0.110	1.03	0.99-1.06	0.983	1.00	0.94 - 1.07
**Tumor size**									
pTstage 1	0.056	Reference		0.078	Reference		Group size too small / too few events		
pTstage 2		1.53	0.68 - 3.45		1.44	1.48 - 5.57			
pTstage 3 & 4		3.76	1.32 - 10.74		3.71	0.26-14.56			
**Lymph node stage**									
pNstage 1 & 2	0.005	Reference		0.154	Reference		0.008	Reference	
pNstage 3		2.52	1.38 - 4.60		1.71	0.84 - 3.46		5.84	1.69-20.21
**Lymphovascular invasion**	0.194	1.54	0.78 - 3.03	0.459	1.33	0.61 - 2.92	0.407	1.33	0.34 - 5.15
**HER2 status**	1.000	1.00	0.56 - 1.80	0.538	0.81	0.41 - 1.59	0.407	1.69	0.49 - 5.85
**sTILs**				\			\		
20% cut off	<0.001	0.26	0.13 - 0.51						
Increments	<0.001	0.96	0.93 - 0.98						
**TLS**	0.002	0.25	0.09 - 0.71	0.010	0.23	0.05 - 0.94	0.355	0.50	0.11-2.37

LN characteristics	Cancer-free LN			Involved LN			Cancer-free LN (<20% sTILs)			Cancer-free LN (≥20% sTILs)		
**Total number of GCs across all assessed LNs per patient**	**Model P**	**HR**	**CI**	**Model P**	**HR**	**CI**	**Model P**	**HR**	**CI**	**Model P**	**HR**	**CI**
Total GCs number	0.769	1.00	0.99-1.01	0.459	1.00	0.98-1.01	0.427	0.99	0.97 - 1.01	0.019	1.01	1.00 - 1.02
Total GCs number (≤2 / >2)	<0.001	0.33	0.19-0.59	0.023	0.48	0.26-0.88	0.002	0.36	0.19 - 0.69	0.804	1.29	0.16-10.16

Multivariate	Corrected for: Age, pTstage, pNstage, LVI, sTILs & TLS								Corrected for: Age, pTstage, pNstage, LVI & TLS							
**LN characteristics**	Cancer-free LN				Involved LN				Cancer-free LN (<20% sTILs)				Cancer-free LN (≥20% sTILs)			
Across all assessed LNs per patient	**Covariate P**	**Model P**	**HR**	**CI**	**Covariate P**	**Model P**	**HR**	**CI**	**Covariate P**	**Model P**	**HR**	**CI**	**Covariate P**	**Model P**	**HR**	**CI**
Total GCs number	0.010	<0.001	1.01	1.00 - 1.02	0.362	<0.001	1.00	0.99 - 1.01	0.832	0.025	1.00	0.98-1.02	0.086	0.013	1.01	1.00-1.03
Total GCs number (≤2 / >2)	0.110	<0.001	0.58	0.30 - 1.12	0.161	<0.001	0.63	0.33 - 1.20	0.023	0.004	0.41	0.19 -0.89	0.949	0.034	0.93	0.11-7.93

Supplementary Table 2b. Univariate and Multivariate Cox regression analyses for dDFS

dDFS									
**Univariate**	**All cases**			**<20% sTILs**			**≥20% sTILs**		
**Clinicopathological features**	**Model P**	**HR**	**CI**	**Model P**	**HR**	**CI**	**Model P**	**HR**	**CI**
**Age at diagnosis**	0.055	1.03	1.00 - 1.06	0.041	0.103	1.00-	0.949	1.00	0.93 - 1.07
**Tumor size**									
pTstage 1	0.017	Reference		0.034	Reference		Group size too small / too few events		
pTstage 2		1.95	0.76 - 5.03		1.76	0.53 - 5.84			
pTstage 3 & 4		5.43	1.72 - 17.13		5.12	1.32 -19.88			
**Lymph node stage**									
pNstage 1 & 2	0.003	Reference		0.074	Reference		0.018	Reference	
pNstage 3		2.75	1.46 - 5.18		1.99	0.97 - 4.08		5.88	1.47-23.55
**Lymphovascular invasion**	0.219	1.54	0.76 - 3.13	0.324	1.50	0.65 - 3.44	0.953	0.95	0.23 - 4.01
**HER2 status**	0.953	0.98	0.53 - 1.83	0.512	0.79	0.39 - 1.60	0.437	0.44	0.43 - 6.96
**sTILs**									
20% cut off	<0.001	0.22	0.10 - 0.48	\			\		
Increments	<0.001	0.96	0.93 - 0.98						
**TLS**	0.001	0.21	0.06 - 0.67	0.013	0.23	0.06 - 0.98	0.183	0.18	0.04 - 2.38

**LN characteristic**	**Cancer-free LN**			**Involved LN**			**Cancer-free LN**			**Cancer-free LN**		
Across all assessed LNs per patient	**Model P**	**HR**	**CI**	**Model P**	**HR**	**CI**	**Model P**	**HR**	**CI**	**Model P**	**HR**	**CI**
Total GCs number	0.618	1.00	0.99 - 1.01	0.253	0.98	0.98 - 1.01	0.483	0.99	0.97 - 1.01	0.009	1.02	1.01 - 1.03
Total GCs number (≤2 / >2)	<0.001	0.26	0.14 - 0.48	0.038	0.25	0.25 - 0.93	<0.001	0.28	0.14 - 0.56	0.986	1.02	0.13 - 8.29

**Multivariate**	**Corrected for: Age, pTstage, pNstage, LVI, sTILs & TLS**								**Corrected for: Age, pTstage, pNstage, LVI,& TLS**							
**LN characteristics**	**Cancer-free LN**				**Involved LN**				**Cancer-free LN**				**Cancer-free LN**			
Across all assessed LNs per patient	**Covariate P**	**Model P**	**HR**	**CI**	**Covariate P**	**Model P**	**HR**	**CI**	**Covariate P**	**Model P**	**HR**	**CI**	**Covariate P**	**Model P**	**HR**	**CI**
Total GCs number	0.002	<0.001	1.01	1.00 - 1.02	0.565	<0.001	1.00	0.99 - 1.01	0.665	0.005	1.00	0.98 - 1.03	0.044	0.007	1.02	1.00 - 1.03
Total GCs number (≤2 / >2)	0.033	<0.001	0.47	0.23 - 0.94	0.21	<0.001	0.64	0.32 - 1.28	0.009	<0.001	0.34	0.17 - 0.77	0.665	0.031	0.61	0.07 – 5.64

Supplementary Table 2c. Univariate and Multivariate Cox regression analyses for OS

OS

Univariate	All cases			<20% sTILs			≥20% sTILs		
**Clinicopathological features**	**Model P**	**HR**	**CI**	**Model P**	**HR**	**CI**	**Model P**	**HR**	**CI**
**Age at diagnosis**	0.019	1.04	1.01 - 1.08	0.018	1.04	1.01-1.08	0.711	1.02	0.93 - 1.12
**Tumor size**									
pTstage 1	0.004	Reference		0.053	Reference		Group size too small / too few events		
pTstage 2		4.23	1.00 - 17.84		2.30	0.54 - 9.82			
pTstage 3 & 4		10.80	2.18 - 53.54		5.96	1.20 -29.69			
**Lymph node stage**									
pNstage 1 & 2	0.001	Reference		0.087	Reference		0.001	Reference	
pNstage 3		3.34	1.69 - 6.63		2.01	0.94 - 4.34		22.18	2.47-198.7
**Lymphovascular invasion**	0.054	2.22	0.92 - 5.37	0.429	1.42	0.58 - 3.49	Group size too small / too few events		
**HER2 status**	0.564	1.22	0.62 - 2.40	0.980	0.99	0.47 - 2.08	0.310	2.50	0.42 -14.96
**sTILs**				\			\		
20% cut off	<0.001	0.17	0.06 - 0.43						
Increments	<0.001	0.94	0.91 - 0.98						
**TLS**	<0.001	0.08	0.01 - 0.59	0.005	0.13	0.02 - 0.97	Group size too small / too few events		

LN characteristics	Cancer-free LN			Involved LN			Cancer-free LN			Cancer-free LN		
**Total number of GCs across**	**Model P**	**HR**	**CI**	**Model P**	**HR**	**CI**	**Model P**	**HR**	**CI**	**Model P**	**HR**	**CI**
Total GCs number	0.951	1.00	0.99-1.01	0.488	1.00	0.98 - 1.01	0.869	1.00	0.98 - 1.02	0.152	1.01	1.00 - 1.03
Total GCs number (≤2 / >2)	<0.001	0.28	0.14 - 0.55	0.033	0.44	0.22 - 0.91	0.006	0.36	0.17 - 0.75	0.652	0.59	0.07 - 5.25

Multivariate	Corrected for: Age, pTstage, pNstage, LVI, sTILs & TLS								Corrected for: Age, pTstage, pNstage, LVI & TLS							
**LN characteristics**	Cancer-free LN				Involved LN				Cancer-free LN				Cancer-free LN			
Across all assessed LNs per patient	**Covariate P**	**Model P**	**HR**	**CI**	**Covariate P**	**Model P**	**HR**	**CI**	**Covariate P**	**Model P**	**HR**	**CI**	**Covariate P**	**Model P**	**HR**	**CI**
Total GCs number	0.005	<0.001	1.02	1.00 - 1.03	0.092	<0.001	1.01	1.00 - 1.02	0.163	0.002	1.01	1.00 - 1.03	0.372	<0.001	0.96	0.96 - 1.02
Total GCs number (≤2 / >2)	0.351	<0.001	0.69	0.32 - 1.50	0.193	<0.001	0.60	0.28 - 1.29	0.106	0.001	0.48	0.20 – 1.17	Group size too small / too few events			

Supplementary Table 3. Univariate and Multivariate Cox regression analyses for iDFS, dDFS and OS of TNBC and HER2-positive subgroups

Supplementary Table 3a. Univariate and Multivariate Cox regression analyses for iDFS of TNBC and HER2-positive subgroups

	iDFS					
**Univariate**	**TNBC**			**HER2**		
**Clinicopathological features**	**Model P**	**HR**	**CI**	**Model P**	**HR**	**CI**
**Age at diagnosis**	0.076	1.03	1 - 1.07	0.921	1.00	0.95 - 1.06
**Tumor size**						
pTstage 1	0.260	Reference		0.206	Reference	
pTstage 2		1.47	0.55 - 3.92		1.66	0.38 - 7.38
pTstage 3 & 4		3.16	0.85 - 11.8		4.97	0.83 - 29.83
**Lymph node stage**						
pNstage 1 & 2	0.043	Reference		0.049	Reference	
pNstage 3		2.48	1.1 - 5.6		2.64	1.04 - 6.7
**Lymphovascular invasion**	0.218	1.64	0.73 - 3.71	0.616	1.36	0.39 - 4.7
**sTILs**						
20% cut off	<0.001	0.19	0.07 - 0.49	0.064	0.40	0.14 - 1.11
Increments	0.002	0.96	0.93 - 0.99	0.010	0.96	0.92 - 1
**TLS**	0.010	0.22	0.05 - 0.92	0.065	0.30	0.07 - 1.32

**LN characteristics**	**Cancer-free LN**			**Involved LN**			**Cancer-free LN**			**Involved LN**		
	**Model P**	**HR**	**CI**	**Model P**	**HR**	**CI**	**Model P**	**HR**	**CI**	**Model P**	**HR**	**CI**
Total GCs number	0.161	1.01	1 - 1.02	0.740	1.00	0.99 - 1.01	0.023	0.95	0.9 - 1.01	0.079	0.98	0.94 - 1.01
Total GCs number (≤2 / >2)	<0.001	0.25	0.12 - 0.52	0.004	0.32	0.15 - 0.67	0.139	0.49	0.19 - 1.24	0.849	1.13	0.32 - 3.96

**Multivariate**	**Corrected for: Age, pTstage, pNstage, LVI, sTILs & TLS**								**Corrected for: Age, pTstage, pNstage, LVI, sTILs & TLS**							
**LN characteristics**	**Cancer-free LN**				**Involved LN**				**Cancer-free LN**				**Involved LN**			
Across all assessed LNs per patient	**Covariate P**	**Model**	**HR**	**CI**	**Covariate P**	**Model P**	**HR**	**CI**	**Covariate P**	**Model P**	**HR**	**CI**	**Covariate P**	**Model P**	**HR**	**CI**
Total GCs number	<0.001	<0.001	1.01	1.01 - 1.03	0.110	<0.001	1.00	1.00 - 1.02	0.127	0.046	0.94	0.87 – 1.02	0.260	0,169	0.93	0.93 – 1.02
Total GCs number (≤2 / >2)	0.017	<0.001	0.37	0.16 – 0.84	0.092	<0.001	0.50	0.22 - 1.12	0.959	0.144	0.97	0.26 – 3.59	0.713	0.261	1.27	0.35 – 4.63

Supplementary Table 3b. Univariate and Multivariate Cox regression analyses for dDFS of TNBC and HER2-positive subgroups

	dDFS					
**Univariate**	**TNBC**			**HER2**		
**Clinicopathological features**	**Model P**	**HR**	**CI**	**Model P**	**HR**	**CI**
**Age at diagnosis**	0.035	1.04	1 - 1.08	0.780	1.01	0.95 - 1.07
**Tumor size**						
pTstage 1	0.082	Reference		0.168	Reference	
pTstage 2		2.37	0.7 - 8.02		1.37	0.30 - 6.20
pTstage 3 & 4		5.41	1.21 - 24.21		5.11	0.85 - 30.71
**Lymph node stage**						
pNstage 1 & 2	0.021	Reference		0.060	Reference	
pNstage 3		2.90	1.26 - 6.69		2.68	0.99 - 7.21
**Lymphovascular invasion**	0.404	1.42	0.62 - 3.26	0.310	2.02	0.46 - 8.90
**sTILs**						
20% cut off	<0.001	0.16	0.06 - 0.47	0.051	0.35	0.11 - 1.09
increments	0.002	0.96	0.93 - 0.99	0.012	0.95	0.91 - 1.00
**TLS**	0.022	0.25	0.06 - 1.05	0.018	0.16	0.02 - 1.19

**LN characteristics**	**Cancer-free LN**			**Involved LN**			**Cancer-free LN**			**Involved LN**		
	**Model P**	**HR**	**CI**	**Model P**	**HR**	**CI**	**Model P**	**HR**	**CI**	**Model P**	**HR**	**CI**
Total GCs number	0.120	1.01	1.00 - 1.02	0.962	1.00	0.99 - 1.01	0.015	0.94	0.88 - 1.01	0.024	0.96	0.91 - 1.01
Total GCs number (≤2 / >2)	<0.001	0.20	0.09 - 0.44	0.015	0.36	0.17 - 0.79	0.057	0.38	0.14 - 1.01	0.910	0.93	0.26 - 3.33

**Multivariate**	**Corrected for: Age, pTstage, pNstage, LVI, sTILs & TLS**								**Corrected for: Age, pTstage, pNstage, LVI, sTILs & TLS**							
**LN characteristics**	**Cancer-free LN**				**Involved LN**				**Cancer-free LN**				**Involved LN**			
Across all assessed LNs per patient	**Covariate P**	**Model**	**HR**	**CI**	**Covariate P**	**Model P**	**HR**	**CI**	**Covariate P**	**Model P**	**HR**	**CI**	**Covariate P**	**Model P**	**HR**	**CI**
Total GCs number	<0.001	<0.001	1.02	1.01 - 1.03	0.429	<0.001	1.01	0.99 - 1.02	0.200	0.011	0.96	0.90 - 1.02	0.089	0.007	0.95	0.90 – 1.01
Total GCs number (≤2 / >2)	0.004	<0.001	0.29	0.13 – 0.67	0.034	<0.001	0.43	0.20 – 0.94	0.375	0.037	0.60	0.2 - 1.84	0.970	0.069	0.98	0.27 – 3.57

Supplementary Table 3c. Univariate and Multivariate Cox regression analyses for OS of TNBC and HER2-positive subgroups

	OS					
**Univariate**	**TNBC**			**HER2**		
**Clinicopathological features**	**Model P**	**HR**	**CI**	**Model P**	**HR**	**CI**
**Age at diagnosis**	0.012	1.06	1.01- 1.11	0.714	1.01	0.95 - 1.07
**Tumor size**						
pTstage 1	0.040	Reference		0.095	Reference	
pTstage 2		5.50	0.73 - 41.63		2.91	1.01 - 22.56
pTstage 3 & 4		11.39	1.18 - 109.58		9.74	0.38 - 93.94
**Lymph node stage**						
pNstage 1 & 2	0.009	Reference		0.052	Reference	
pNstage 3		3.89	1.53 - 9.89		2.84	1.03 - 7.85
**Lymphovascular invasion**	0.267	1.74	0.63 - 4.84	0.086	4.22	0.55 - 32.1
**sTILs**						
20% cut off	<0.001	0.11	0.03 - 0.47	0.019	0.26	0.07 - 0.91
increments	0.002	0.95	0.9 - 0.99	0.004	0.94	0.89 - 0.99
**TLS**	Group size too small / too few events			0.026	0.17	0.02 - 1.28

**LN characteristics**	**TNBC Cancer-free LN**			**TNBC Involved LN**			**HER2 Cancer-free LN**			**HER2 Involved LN**		
**Total number of GCs across all assessed LNs per patient**	**Model P**	**HR**	**CI**	**Model P**	**HR**	**CI**	**Model P**	**HR**	**CI**	**Model P**	**HR**	**CI**
Total GCs number	0.301	1.01	1.00 - 1.02	0.565	1.00	0.99 - 1.02	0.019	0.94	0.88 - 1.01	0.027	0.96	0.91 - 1.01
Total GCs number (≤2 / >2)	0.004	0.24	0.10 - 0.60	0.013	0.31	0.12 - 0.75	0.036	0.33	0.12 - 0.92	0.848	0.88	0.24 - 3.20

**Multivariate**	**TNBC: Corrected for: Age, pTstage, pNstage, LVI, sTILs & TLS**								**HER2: Corrected for: Age, pTstage, pNstage, LVI, sTILs & TLS**							
**LN characteristics**	**Cancer-free LN**				**Involved LN**				**Cancer-free LN**				**Involved LN**			
Across all assessed LNs per patient	**Covariate P**	**Model P**	**HR**	**CI**	**Covariate P**	**Model P**	**HR**	**CI**	**Covariate P**	**Model P**	**HR**	**CI**	**Covariate P**	**Model P**	**HR**	**CI**
Total GCs number	0.002	<0.001	1.02	1.01 - 1.03	0.080	<0.001	1.01	1.00 - 1.03	0.401	0.015	0.96	0.88 - 1.05	0.236	0.058	0.96	0.91 - 1.02
Total GCs number (≤2 / >2)	0.119	<0.001	0.46	0.17 - 1.22	0.077	<0.001	0.40	0.15 – 1.10	0.851	0.021	1.14	0.28 - 4.72	0.751	0.102	1.24	0.33 - 4.71

Supplementary Table 4. Multivariate Cox regression analyses for iDFS, dDFS and OS comparing the prognostic value of the proportion of LNs with GCs and the ratio of GCs in LNs

Multivariate analysis									
corrected for Age, pTstage, pNstage, LVI & TLS									
	<20% sTILs					≥20% sTILs			
Covariate	Covariate P value	Model P values	HR	CI		Covariate P value	Model P values	HR	CI
iDFS									
Proportion of LNs with GC (Involved and cancer-free LNs)	0.236	0.015	0.20	0.01 - 2.88		0.058	0.010	33.34	0.89 - 1255.32
Proportion of LNs with GC (cancer-free LNs)	0.607	0.023	0.54	0.05 - 5.58		0.037	0.007	14.92	1.18 - 187.97
Proportion of LNs with GC (Involved LNs)	0.136	0.021	0.47	0.17 - 1.27		0.490	0.039	0.44	0.04 - 4.55
Total GCs / Total LNs (Involved and cancer-free)	0.938	0.026	0.99	0.73 - 1.33		0.069	0.012	1.20	0.99 - 1.46
Total GCs / Total LNs (cancer-free)	0.823	0.025	0.96	0.64 - 1.42		0.009	0.004	1.37	1.08 - 1.73
Total GCs / Total LNs (Involved)	0.250	0.026	0.95	0.87 - 1.04		0.603	0.041	0.98	0.93 - 1.04
dDFS									
Proportion of LNs with GC (Involved and cancer-free LNs)	0.129	0.002	0.10	0.01 - 1.94		0.040	0.007	66.23	1.22 - 3602.85
Proportion of LNs with GC (cancer-free LNs)	0.542	0.004	0.46	0.04 - 5.49		0.024	0.004	32.03	1.58 - 650.92
Proportion of LNs with GC (Involved LNs)	0.095	0.004	0.40	0.13 - 1.17		0.844	0.040	0.77	0.05 - 10.95
Total GCs / Total LNs (Involved and cancer-free)	0.585	0.004	0.90	0.62 - 1.31		0.068	0.012	1.20	0.99 - 1.46
Total GCs / Total LNs (cancer-free)	0.890	0.005	0.97	0.64 - 1.47		0.025	0.006	1.33	1.04 - 1.70
Total GCs / Total LNs (Involved)	0.034	0.001	0.87	0.76 - 0.99		0.143	0.011	0.85	0.69 - 1.06
OS									
Proportion of LNs with GC (Involved and cancer-free LNs)	0.765	0.003	0.63	0.03 - 13.42		0.443	<0.001	0.05	0 - 116.27
Proportion of LNs with GC (cancer-free LNs)	0.470	0.003	2.51	0.21 - 30.25		0.521	<0.001	0.19	0 - 30.14
Proportion of LNs with GC (Involved LNs)	0.154	0.005	0.42	0.13 - 1.39		0.541	<0.001	0.25	0 - 20.97
Total GCs / Total LNs (Involved and cancer-free)	0.602	0.003	1.10	0.78 - 1.55		0.431	<0.001	0.80	0.46 - 1.39
Total GCs / Total LNs (cancer-free)	0.305	0.002	1.20	0.85 - 1.70		0.424	<0.001	0.80	0.46 - 1.39
Total GCs / Total LNs (Involved)	0.066	0.002	0.88	0.76 - 1.01		0.677	<0.001	0.93	0.65 - 1.32

**Multivariate analysis**									
**corrected for Age, pTstage, pNstage, LVI, TLS & sTILs**									
	**TNBC**					**HER2**			
**Covariate**	**Covariate P value**	**Model P values**	**HR**	**CI**		**Covariate P value**	**Model P values**	**HR**	**CI**
**iDFS**									
Proportion of LNs with GC (Involved and cancer-free LNs)	0.178	0.001	4.41	0.51 - 38.29		0.547	0.129	0.37	0.01 - 9.5
Proportion of LNs with GC (cancer-free LNs)	0.055	<0.001	5.77	0.96 - 34.56		0.734	0.139	0.57	0.02 - 14.08
Proportion of LNs with GC (Involved LNs)	0.212	0.001	0.50	0.17 - 1.48		0.826	0.268	1.23	0.2 - 7.55
Total GCs / Total LNs (Involved and cancer-free)	0.002	<0.001	1.25	1.08 - 1.45		0.277	0.092	0.76	0.46 - 1.25
Total GCs / Total LNs (cancer-free)	<0.001	<0.001	1.41	1.17 - 1.7		0.699	0.137	0.91	0.57 - 1.46
Total GCs / Total LNs (Involved)	0.692	0.001	0.98	0.91 - 1.07		0.719	0.261	0.99	0.94 - 1.04
**dDFS**									
Proportion of LNs with GC (Involved and cancer-free LNs)	0.124	<0.001	5.99	0.61 - 58.74		0.154	0.029	0.07	0 - 2.74
Proportion of LNs with GC (cancer-free LNs)	0.030	<0.001	8.08	1.23 - 53.1		0.588	0.054	0.38	0.01 - 12.86
Proportion of LNs with GC (Involved LNs)	0.356	<0.001	0.58	0.19 - 1.83		0.549	0.156	0.55	0.08 - 3.88
Total GCs / Total LNs (Involved and cancer-free)	0.003	<0.001	1.27	1.08 - 1.49		0.073	0.015	0.52	0.25 - 1.06
Total GCs / Total LNs (cancer-free)	<0.001	<0.001	1.46	1.19 - 1.78		0.124	0.021	0.48	0.19 - 1.23
Total GCs / Total LNs (Involved)	0.186	<0.001	0.91	0.8 - 1.04		0.228	0.078	0.92	0.8 - 1.05
**OS**									
Proportion of LNs with GC (Involved and cancer-free LNs)	0.109	6.80E-06	9.83	0.6 - 161.44		0.530	0.02	0.30	0.01 - 13.32
Proportion of LNs with GC (cancer-free LNs)	0.023	2.51E-06	14.40	1.45 - 142.92		0.747	0.02	1.87	0.04 - 82.66
Proportion of LNs with GC (Involved LNs)	0.286	1.35E-05	0.49	0.13 - 1.82		0.604	0.10	0.58	0.08 - 4.47
Total GCs / Total LNs (Involved and cancer-free)	0.020	2.29E-06	1.29	1.04 - 1.59		0.298	0.01	0.71	0.37 - 1.36
Total GCs / Total LNs (cancer-free)	0.004	1.18E-06	1.44	1.12 - 1.85		0.454	0.02	0.73	0.33 - 1.65
Total GCs / Total LNs (Involved)	0.490	1.81E-05	0.95	0.81 - 1.1		0.302	0.06	0.93	0.82 - 1.06
