# Supplementary material for: Systemic immune reaction in axillary lymph nodes adds to tumor-infiltrating lymphocytes in triple-negative breast cancer prognostication
Source: NPJ Breast Cancer. 2021 Jul 5;7:86. doi: 10.1038/s41523-021-00292-y (PMC8257702; doi:10.1038/s41523-021-00292-y)
Supplement: Supplementary file 3 — Reporting Summary [file 41523_2021_292_MOESM3_ESM.pdf]

nature research

Corresponding author(s): Anita Grigoriadis

Last updated by author(s): May 6, 2021

Reporting Summary

Nature Research wishes to improve the reproducibility of the work that we publish. This form provides structure for consistency and transparency in reporting. For further information on Nature Research policies, see our Editorial Policies and the Editorial Policy Checklist.

Statistics

For all statistical analyses, confirm that the following items are present in the figure legend, table legend, main text, or Methods section.

n/a	Confirmed	
☐	☒	The exact sample size (*n*) for each experimental group/condition, given as a discrete number and unit of measurement
☒	☐	A statement on whether measurements were taken from distinct samples or whether the same sample was measured repeatedly
☐	☒	The statistical test(s) used AND whether they are one- or two-sided<br>*Only common tests should be described solely by name; describe more complex techniques in the Methods section.*
☐	☒	A description of all covariates tested
☐	☒	A description of any assumptions or corrections, such as tests of normality and adjustment for multiple comparisons
☐	☒	A full description of the statistical parameters including central tendency (e.g. means) or other basic estimates (e.g. regression coefficient) AND variation (e.g. standard deviation) or associated estimates of uncertainty (e.g. confidence intervals)
☒	☐	For null hypothesis testing, the test statistic (e.g. *F*, *t*, *r*) with confidence intervals, effect sizes, degrees of freedom and *P* value noted<br>*Give P values as exact values whenever suitable.*
☒	☐	For Bayesian analysis, information on the choice of priors and Markov chain Monte Carlo settings
☒	☐	For hierarchical and complex designs, identification of the appropriate level for tests and full reporting of outcomes
☒	☐	Estimates of effect sizes (e.g. Cohen's *d*, Pearson's *r*), indicating how they were calculated

Our web collection on statistics for biologists contains articles on many of the points above.

Software and code

Policy information about availability of computer code

Data collection	Qupath for H&E annotation
Data analysis	R (Version 3.5.1) for statistical and outcome analysis

For manuscripts utilizing custom algorithms or software that are central to the research but not yet described in published literature, software must be made available to editors and reviewers. We strongly encourage code deposition in a community repository (e.g. GitHub). See the Nature Research guidelines for submitting code & software for further information.

Data

Policy information about availability of data

All manuscripts must include a data availability statement. This statement should provide the following information, where applicable:

- Accession codes, unique identifiers, or web links for publicly available datasets
- A list of figures that have associated raw data
- A description of any restrictions on data availability

De-identified data, including clinical covariates are available in supplementary files. Code available upon request.

Field-specific reporting

Please select the one below that is the best fit for your research. If you are not sure, read the appropriate sections before making your selection.

☒ Life sciences ☐ Behavioural & social sciences ☐ Ecological, evolutionary & environmental sciences

For a reference copy of the document with all sections, see nature.com/documents/nr-reporting-summary-flat.pdf

Life sciences study design

All studies must disclose on these points even when the disclosure is negative.

Sample size	161 HR-negative patients were available with clinical data matching the criteria of this study. Patients were decided based on HER2 status (positive n=62 and negative n=99) and the number of germinal centers in their cancer-free lymph nodes (≤2 GCs n = 40 and >2 GCs n = 120)
Data exclusions	One patient was excluded. This was due to the staining of their sections being too dark for analysis.
Replication	N/A
Randomization	N/A
Blinding	N/A

Reporting for specific materials, systems and methods

We require information from authors about some types of materials, experimental systems and methods used in many studies. Here, indicate whether each material, system or method listed is relevant to your study. If you are not sure if a list item applies to your research, read the appropriate section before selecting a response.

Materials & experimental systems

n/a	Involved in the study	
☒	☐	Antibodies
☒	☐	Eukaryotic cell lines
☒	☐	Palaeontology and archaeology
☒	☐	Animals and other organisms
☐	☒	Human research participants
☐	☒	Clinical data
☒	☐	Dual use research of concern

Methods

n/a	Involved in the study	
☒	☐	ChIP-seq
☒	☐	Flow cytometry
☒	☐	MRI-based neuroimaging

Human research participants

Policy information about studies involving human research participants

Population characteristics	161 female breast no special type carcinoma patients, treated between 2005 - 2010 at Medical University Cancer Hospital, China. Patients were HR-negative, had positive lymph nodes and had histological grade 3. Median age was 52 with a range of 23 - 75. Treatment protocols were the same for all patients.
Recruitment	Retrospective study using sections stored in hospital's biobank
Ethics oversight	Medical Ethics Committee of Tianjin Medical University Cancer Institute and Hospital, Ek2020021

Note that full information on the approval of the study protocol must also be provided in the manuscript.

Clinical data

Policy information about clinical studies

All manuscripts should comply with the ICMJE guidelines for publication of clinical research and a completed CONSORT checklist must be included with all submissions.

Clinical trial registration	N/A
Study protocol	N/A
Data collection	Patient selection and data analyses are reported according to Reporting Recommendations for Tumor Marker Prognostic Studies (REMARK) criteria.

Outcomes

The primary endpoint was distant Disease Free Survival, defined as the date of first distant recurrence or death from any cause. Invasive Disease Free Survival was defined as the date of first invasive recurrence, or second primary, or death from any cause
